# Violence in the emergency department: a quantitative survey study of healthcare providers in India

**DOI:** 10.1186/s12245-024-00653-x

**Published:** 2024-07-03

**Authors:** Tania Ahluwalia, Sukhpreet Singh, Navvin Gandhi, Serkan Toy, Katherine Douglass, Janice Blanchard, Kevin Davey

**Affiliations:** 1 Children’s National Health System, Division of Emergency Medicine, 111 Michigan Avenue, Washington, DC 20010 USA; 2 Max Healthcare Saket, Delhi, India; 3 Meenakshi Mission Hospital and Research Center, Madurai, India; 4 Departments of Basic Science Education & Health Systems and Implementation Science, Virginia Tech Carilion School of Medicine, Roanoke, VA USA; 5 Department of Emergency Medicine, George Washington University School of Medicine & Health Sciences, Washington, DC USA

**Keywords:** Healthcare workplace violence, Violence against Healthcare workers, Developing emergency care systems, India

## Abstract

**Background:**

Workplace violence (WPV) in Emergency Departments (EDs) is an increasingly recognized challenge healthcare providers face in low-resource settings. While studies have highlighted the increased prevalence of WPV in healthcare, most of the existing research has been conducted in developed countries with established laws and repercussions for violence against healthcare providers. More data on WPV against ED providers practicing in low-resource settings is necessary to understand these providers’ unique challenges.

**Objective:**

This study aims to gain insight into the incidence and characteristics of WPV among ED healthcare providers in India.

**Methods:**

This study was conducted at two EDs in geographically distinct regions of India. A survey was designed to assess violence in EDs among healthcare providers. Surveys were distributed to ED workplace providers, completed by hand, and returned anonymously. Data was entered and stored in the RedCAP database to facilitate analysis.

**Results:**

Two hundred surveys were completed by physicians, nurses, and paramedics in Indian EDs. Most reported events involved verbal abuse (68%), followed by physical abuse (26%), outside confrontation (17%), and stalking (5%). By far, the most common perpetrators of violence against healthcare workers were bystanders including patient family members or other accompanying individuals. Notably, reporting was limited, with most cases conveyed to ED or hospital administration.

**Conclusion:**

These results underscore the prevalence of WPV among Indian ED healthcare providers. High rates of verbal abuse followed by physical abuse are of concern. Most perpetrators of WPV against healthcare providers in this study were patient family members or bystanders rather than the patients themselves. It is imperative to prioritize implementing prevention strategies to create safer work environments for healthcare workers.

**Supplementary Information:**

The online version contains supplementary material available at 10.1186/s12245-024-00653-x.

## Background

Workplace violence (WPV) against healthcare providers is well-documented and increasingly common [1–4]. The emergency department (ED) is especially susceptible to violence due to stressors, including high patient volume, acuity of illness, rotating staff, and late working hours [5–7]. Previous studies on violence in EDs have predominantly centered on developed countries with established laws and legal repercussions for violence against healthcare providers. Limited research exists in low-resource settings where regulations protecting healthcare providers are less common [8]. In such settings, emergency medicine (EM) is still developing, and clear laws, regulations, and enforcement mechanisms may be lacking in the ED. Patients may misunderstand available care in the ED and feel their needs are not met, potentially further exacerbating the risk of violence against healthcare workers [4]. Existing studies in low-and middle-income countries, including India, often focus on a single type of provider such as physicians, nurses, or paramedics [9–11]. To address violence against emergency healthcare providers in India and resource-limited settings, understanding the issues related to violence in EDs is crucial. Our institution has long-standing partnership programs in EM education and training across India. A recent qualitative study at our partner institutions revealed common themes of violence in the ED including types of violence, experiences of violence, causes of violence, description of violent events, consequences of violence, responsibility for the violence, and proposed prevention strategies [12]. While this study revealed insights into ED providers’ unique challenges in India, its qualitative nature limits its generalizability, prompting a quantitative survey among ED healthcare providers at two Indian emergency departments. This study aims to understand better the issues surrounding WPV experienced by ED providers in India. This multicenter, quantitative study of WPV includes 1st -3rd year EM residents, nurses, paramedics, consultants, technicians, ambulance drivers, and environmental services.

## Methods

This study was conducted at two EDs in geographically distinct regions of India. The study was conducted from June 1 to August 31, 2021. This study followed a qualitative study on WPV in India, which identified themes that served as a template to create a survey to explore further and quantify the recognized themes [12]. An expert panel designed and reviewed a survey and then piloted it with ED healthcare providers before use (see Supplementary File). Based on the pilot, minor revisions were made. The survey was designed to query ED healthcare providers on their own experiences and witnessed experiences of ED WPV. Specifically, the survey addressed four different types of ED WPV: verbal abuse, physical abuse, outside confrontations, and stalking. Outside confrontations are defined as any unpleasant or threatening interaction with a patient, family, or bystanders after providing medical care [4]. Stalking is any unwanted or threatening behavior by the patient or someone persistently following the patient over time [4].

Additionally, the survey asks participants about the frequency of reporting incidents of ED WPV faced by them and the availability of resources to mitigate WPV in their EDs. The questionnaire used content and language appropriate for the target population. Surveys were designed in English and translated into Hindi, Punjabi, and Tamil using an official translator service. During the study, surveys and information sheets were available in all four languages. Surveys were distributed to ED workplace providers by student research assistants. Surveys were completed by hand and returned anonymously via a designated collection box in the ED. No personal identifying information was collected. Every week, research assistants at each institution collected surveys and entered the data into a secure database, RedCap (Research Electronic Data Capture) hosted at GW. The GW Institutional Review Board exempted the study. Chi-square tests were used to compare subgroups of survey respondents (gender, years in practice, and job title) in terms of witnessing, personally experiencing, or reporting verbal abuse, physical abuse, outside confrontation, and stalking. For these analyses, JMP®, Version 16, SAS Institute Inc., Cary, NC, 1989–2023 was used.

## Results

### Demographics

200 hundred workplace providers from two hospital EDs responded to the survey. There were 119 respondents from Site 1 and 81 respondents from Site 2. Most respondents were male (59%; *n* = 118), and 41% (*n* = 82) were female. All respondents were between 20 and 50 years old, with 66% (*n* = 132) between 20 and 30. Most respondents (55%, *n* = 111) were in clinical practice for less than five years. 29% (*n* = 58) were in clinical practice for 5–10 years, 9.5% (*n* = 19) for 11–15 years, and the remainder were in practice for over 16 years. Most respondents were 1st -3rd year EM residents (29.5%, *n* = 59), closely followed by nurses (24.5%, *n* = 49). Staff members such as technicians, ambulance drivers, and environmental services accounted for 18.5% (*n* = 37), and consultants/attendings attributed to 9.5% (*n* = 19). Table 1 lists the demographics of survey respondents.

**Table 1:** Demographics

	Percent	Frequency
**Age** (***n* = 200**)		
20–30	66%	132
31–40	25.5%	51
41–50	8.5%	17
**Years in Clinical Practice** (***n* = 200**)		
< 5 years	55.5%	111
5–10 years	29%	58
11–15	9.5%	19
16–20 years	4%	8
> 20 years	2%	4
**Job Positions** (***n* = 200**)		
1st -3rd year EM residents	29.5%	59
Nurses	24.5%	49
Paramedics	18%	36
Consultants	9.5%	19
Others (technicians, ambulance drivers, environmental services)	18.5%	37

### Verbal abuse

Most respondents (68%; *n* = 136) reported witnessing verbal abuse in the ED. The abusive parties were most often the patient’s family/bystanders (47%; *n* = 93), followed by the patients themselves (39%; *n* = 53)(Table 2). Several respondents (48%, *n* = 127) reported personally experiencing verbal abuse, with 72% (*n* = 92) of abusers being the patient’s family/bystanders (Table 3). Verbal abuse was reported as most commonly occurring monthly (37%; *n* = 73)(Table 4). Only 50% (*n* = 68) of those who witnessed verbal abuse (*n* = 136) reported it, of which reports were made to the ED administrator (62%, *n* = 42), followed by hospital administrators (56%, *n* = 38)(Table 5). Most survey respondents stated that they did not report abuse as they did not experience an incident that was serious enough to report (39%; *n* = 51).

**Table 2:** Types of witnessed abuse

	Verbal abuse (*n* = 200)		Physical abuse (*n* = 200)		Outside confrontation (*n* = 200)	
	%	Frequency	%	Frequency	%	Frequency
Witnessed Abuse	68	136	26	51	17	34
**Of those that witnessed abused, the abusers were**:						
Patient	39	53/136	73	37/51	15	5/34
Patient’s family/bystanders	47	93/136	59	30/51	88	30/34
ED staff members	29	39/136	4	2/51	15	5/34
Hospital staff members	7	9/136	2	1/51	9	3/34
Specialists from other departments	14	19/136	1	3/51	9	3/34
Other	6	8/136	8	4/51	6	2/34

**Table 3:** Types of experienced abuse

	Verbal abuse		Physical abuse		Outside confrontation		Stalking	
	%	Frequency	%	Frequency	%	Frequency	%	Frequency
Respondent was abused	48	127/200	20	40/200	18	35/200	5	18/200
***Of those that were abused, the abusers were***								
Patient	39	49/127	78	31/40	9	3/35	2	3/18
Patient’s family/bystanders	72	92/127	58	23/40	94	33/35	56	10/18
ED staff members	32	41/127	8	3/40	11	4/35	0	0
Hospital staff members	71	9/127	3	1/40		2/35	2	4/18
Specialists from other departments	23	29/127	0	0/40	9	3/35	1	1/18
Other	7	9/127	2	8/40	0	1/35	1	2/18

### Physical abuse

Physical abuse was reported to be witnessed by 26% (*n* = 51) of respondents. Additionally, 20% (*n* = 40) of respondents reported personally experiencing physical abused in the ED. (See Tables 2 and 3). The abusive party was the patient (78%, *n* = 31) for abuse against the respondent.

**Table 4:** Occurrence of abuse (*n* = 200)

	Verbal Abuse		Physical Abuse		Outside Confrontation		Stalking	
	%	Frequency	%	Frequency	%	Frequency	%	Frequency
Daily	9	18	1	2	2	4	1	2
Weekly	2	40	4	8	4	8	2	4
Monthly	37	73	16	31	9	18	4	8
Every six months	13	26	21	42	13	26	8	15
Yearly	6	12	27	53	30	59	32	63
Never	16	31	32	64	43	85	88	106

**Table 5:** Reported and unreported abuse (*n* = 200)

	Verbal Abuse		Physical Abuse		Outside Confrontation		Stalking	
	%	Frequency	%	Frequency	%	Frequency	%	Frequency
Yes	34	68	15	29	9	18	7	13
No	66	132	86	171	91	182	94	187
**Of those who reported abuse, they reported to**:								
Local police	12	8/68	17	5/29	17	3/18	15	2/13
Hospital police	6	4/68	7	2/29	22	4/18	15	2/13
ED administrator	62	42/68	66	19/29	61	11/18	46	6/13
Hospital administrator	56	38/68	55	16/29	33	6/18	38	5/13
Others	28	19/68	24	7/29	11	2/18	0	0/13
**Of those who did not report abuse, it was because**:								
Never experienced an incident that was serious enough to report	39	51/132	36	62/171	26	48/182	23	43/187
Belief that nothing would be done even if reported	24	33/132	9	15/171	14	25/182	4	7/187
Fear of retribution	15	20/132	9	15/171	7	12/182	3	6/187
Never witnessed or experienced abuse	27	35/132	48	82/171	59	107/182	68	128/187
Other	7	9/132	7	12/171	3	5/182	3	6/187

In both situations, after the patient, this was followed by the patient’s family/bystander (59%, *n* = 30 for witnessed abuse; 58%, *n* = 23 for abuse against the respondent). According to respondents, physical abuse occurs in the following order: never (32%, *n* = 64), then yearly (27%, *n* = 53). Of those who reported either witnessing or experiencing physical abuse (59%, *n* = 29 of 51 who witnessed abuse), it was reported to the ED administrators most often (66%, *n* = 19), followed by hospital administrators (55%, *n* = 16). Most respondents (86%; *n* = 171) did not report an incident of personal or witnessed physical abuse. Of those who did not report physical abuse, it was because they did not see or experience physical abuse in the ED (48%, *n* = 82), followed by not experiencing an incident severe enough to report (36%, *n* = 62), the belief that nothing would be done even if said (9%, *n* = 15), fear of retribution (9%, *n* = 15), and other (7%, *n* = 12). A few respondents (5%; *n* = 10) reported experiencing an incident where a weapon was brandished against them.

### Outside confrontation

Of those who reported either witnessing or experiencing outside confrontation (53%, *n* = 18 of 34), it was reported to ED administrators (61%, *n* = 11), followed by hospital administrators (33%; *n* = 6). Most respondents (91%; *n* = 182) did not report an incident from an outside confrontation, which was because respondents did not witness or experience an outside confrontation (59%; *n* = 107), followed by not experiencing an incident that was serious enough to report (26%; *n* = 48) and the belief that nothing would be done even if reported (14%; *n* = 25).

### Stalking

There were no episodes of witnessing stalking. Some respondents (5%; *n* = 18) reported being stalked due to a patient encounter. Of those who were stalked, the stalker was most commonly the patient’s family/bystander (56%; *n* = 10), followed by other hospital staff (2%; *n* = 4) and patients (2%; *n* = 3). Respondents shared that stalking occurs never (88%; *n* = 106), followed by yearly (32%; *n* = 63). 72% (*n* = 13 of 18 who were stalked) reported stalking. Of those who reported stalking (7%, *n* = 11), they reported it to ED administrators most commonly (46%; *n* = 6), followed by hospital administrators (38%; *n* = 5). Reports were not made by 94% (*n* = 187) because respondents have not experienced stalking (68%; *n* = 128), followed by not experiencing an incident that was serious enough to report (23%; *n* = 43).

### Subgroup analyses

#### Gender

Out of those who indicated personally experiencing physical abuse in the ED (*n* = 40 out of 200), the majority, 72.5%, *n* = 29, were male. The likelihood of male respondents experiencing physical abuse was marginally significant, per likelihood ratio, compared to females, X^2^ (1, *N* = 200) = 3.91, *p* = .048). Nineteen of those 40 respondents (47.5%) who reported personally experiencing physical abuse indicated reporting this abuse. No significant difference was noted between male and female gender for reporting personally experienced physical abuse. X^2^ (1, *N* = 40) = 2.57, *p* > .05. However, when all respondents were included, 29 of them (out of 200) indicated reporting physical abuse; male respondents were more likely to report such abuse, X^2^ (1, *N* = 200) = 6.23, *p* = .01. Gender was not a factor in experiencing other types of abuse or reporting.

#### Years in practice

Except for witnessed physical abuse and stalking in the ED, all other abuse types were found to be significant *p* < .05, indicating an increased incidence of witnessing or experiencing abuse with increased years in practice. See Table 6 for crosstabs of abuse by year in practice.

**Table 6:** Abuse by years in practice

Years in practice	Verbal witness *N* (%)		Verbal personal *N* (%)		Physical witness *N* (%)		Physical personal *N* (%)		Outside confrontation *N* (%)		Stalking *N* (%)	
	No	Yes	No	Yes	No	Yes	No	Yes	No	Yes	No	Yes
< 5	43 (38.7)	68 (61.3)	52 (46.8)	59 (52.3)	87 (78.4)	24 (21.6)	95 (85.6)	16 (14.4)	98 (88.3)	13 (11.7)	101 (91)	10 (9)
5–10	16 (27.6)	42 (72.4)	15 (25.9)	43 (74.1)	44 (75.9)	14 (24.1)	47 (81)	11 (19)	45 (77.6)	13 (22.4)	54 (93.1)	4 (6.9)
11–15	4 (21.1)	15 (78.9)	5 (26.3)	14 (73.7)	11 (57.9)	8 (42.1)	12 (63.2)	7 (36.8)	15 (78.9)	4 (21.1)	17 (89.5)	2 (10.5)
16–20	0 (0)	8 (100)	0 (0)	8 (100)	5 (62.5)	3 (37.5)	4 (50)	4 (50)	3 (37.5)	5 (62.5)	6 (75)	2 (25)
> 20	1 (25)	3 (75)	1 (25)	3 (75)	2 (50)	2 (50)	2 (50)	2 (50)	4 (100)	0 (0)	4 (100)	0 (0)

In terms of the abusive party, there was a significant pattern that the participants with increased years of practice reported significantly more incidence of experiencing verbal abuse by the patient’s family or bystanders, X^2^ (4, *N* = 200) = 12.20, *p* = .02. In terms of the pairwise differences, those with 5–10 years in practice were more likely to experience verbal abuse by the patient’s family or bystanders than those with less than five years of experience (*p* = .04).

A similar statistically significant pattern was observed for personally experiencing physical abuse. The participants with increased years of practice reported significantly more incidence of experiencing physical abuse by the patient’s family or bystanders, X^2^ (4, *N* = 200) = 12.37, *p* = .02.

For the pairwise differences, those with 11–16 years in practice were more likely to experience physical abuse by the patient’s family or bystanders than those with 5–10 years in practice (*p* = .01).

#### Job title

No significant differences were noted based on the job title. We also examined if the participants differed in terms of experiencing verbal or physical abuse in terms of the abusive party and found no differences.

#### Resource provision after reporting violence

Resources include materials provided to those who reported WPV. 23% (*n* = 45) of respondents stated that resources were available when reporting WPV. Some reported (12%; *n* = 24) that the hospital supplied resources, 7% (*n* = 13) reported that family/friends helped get resources, and 3% (*n* = 6) said that the police provided resources.

#### Security measures in the ED

Security measures available in the ED include hospital security assigned to the ED (79%; *n* = 157), unarmed security officers (23%; *n* = 46), hospital security that can be called to the ED but not stationed there (18%; *n* = 36), screening visitors for weapons (11%; *n* = 22), walk through metal detectors (6.5%; *n* = 13), armed security officers (5%; *n* = 10), police (2%; *n* = 3), handheld metal detectors (1%; *n* = 2), whereas others had no security staff (1%; *n* = 2).

#### Impact of violence in the workplace

30% (*n* = 60) of the respondents stated that incidents of WPV have made them less satisfied with their jobs. Many reported losing sleep (27%; *n* = 53), and some were even afraid to go to work due to incidents of WPV (19%; *n* = 38). A few reported missing work due to a violent incident (10%; *n* = 20). As a result of WPV, respondents have considered leaving their current hospital position (18%; *n* = 36), considered leaving the practice of EM (16%; *n* = 31), sought psychological counseling/support (14%; 27), sought to obtain personal protection such as weapons and pepper spray (9%; *n* = 17), left a previous hospital position (6%; *n* = 11), sought legal counseling/support (4%; *n* = 8), and sought help from police (3%; *n* = 6).

#### WPV and the COVID-19 pandemic

53% (*n* = 106) of respondents reported that the COVID-19 pandemic affected the incidence of WPV in their ED, with 68% (72/106) reporting more WPV.

## Discussion

These results suggest that WPV against healthcare providers is unfortunately common in Indian EDs. Most healthcare providers personally experienced and witnessed other providers experience WPV in the ED. The demographic profile of 200 participating healthcare providers is diverse including EM residents, nurses, paramedics, technicians, ambulance drivers, environmental services, and consultants who have experienced ED WPV firsthand. A significant portion of participants were early in their clinical practice, with 65% having less than five years of experience and 66% between 20 and 30, suggesting that these results cannot be attributed to a longer career in a high-stress setting like the ED. Verbal abuse and outside confrontation were found to be statistically significant for those with more years in practice. Also, those with 5–10 years in practice, were more likely to experience verbal abuse by the patient’s family or bystanders compared to those with less than five years. This suggests that there may be factors associated with mid-career experience levels that increase vulnerability to verbal abuse, such as increased exposure to challenging patient situations. This contradicts studies that found that less experienced employees were exposed to more verbal and physical abuse [13, 14].

Violence in the ED, ranked from most to least common, includes verbal abuse, physical abuse, outside confrontation, and stalking. Verbal abuse was predominantly initiated by the patient’s family members or bystanders, which mirrors findings in Indian studies on WPV against doctors, nurses, and emergency medical technicians [15–21]. This aligns with low-resource settings, where bystanders are the most common perpetrators leading to WPV against doctors [13, 14, 16]. This marks a significant difference from studies of ED WPV conducted in high-resource settings, where patients are the primary perpetrators of ED WPV [5]. This also represents a unique challenge for healthcare providers working in this setting, as violence perpetrated by patient bystanders is complicated by the lack of tools available to mitigate that violence. While violent, intoxicated, or mentally unstable patients can be restrained and sedated, violent or agitated family members frequently can not.

Physical abuse, though less frequent than verbal abuse, is still common. Patients were most commonly identified as the perpetrators, followed by the patient’s family/bystanders. Outside confrontations were most commonly instigated by the patient’s family/bystanders, followed by ED staff members. The least common type of abuse was stalking, most commonly initiated by the patient’s family/bystander. In this study, we found that WPV is often instigated by patients’ bystanders as the most common perpetrators of violence for verbal abuse, outside confrontation, and stalking. This is similar to our prior qualitative study, which found that the involvement of family members/bystanders in violent events was 51.2% [12]. This stands in stark contrast to studies of ED WPV conducted in high-resource settings, which have shown patients directly involved in up to 90% of all violent events, with family or friends only involved in 11% of violent events [5]. The reasons for this difference remain unclear and should be the subject of future research.

A notable finding is that half of the respondents did not report abuse; intriguingly, 39% refrained from reporting because they perceived the incident was not severe enough to report. Previous studies found that WPV is being underreported [13, 22]. This was often due to concern that no change would occur even if reported [13]. The underreporting of any form of abuse may play a role in WPV. Interestingly, 86% of respondents did not report physical abuse, mirroring the trend observed with verbal abuse; 36% did not experience an incident thought to be severe enough to report. This suggests a potential normalization of violence within the ED. Those reporting abuse did so to the ED administrators, followed by hospital administrators. Only 12–17% reported to local police, and 6–11% reported to hospital police.

This study found that less than a quarter of respondents reported that resources were provided when reporting WPV, a concerning issue when considering the underreporting of such incidents. More education should be provided that any form of abuse is not accepted and should be reported. Promoting a reporting culture and open dialogue regarding WPV should be a priority.

The most common security measure available in the ED was hospital security assigned to the ED. Additional security measures included hospital security that can be called to the ED but not stationed there, unarmed security officers, screening visitors for weapons, walk-through metal detectors, armed security officers, police, and handheld metal detectors.

This study also highlights the impact of WPV on job satisfaction and mental health, as respondents reported losing sleep, fear of going to work, and missing work due to experiences of WPV. Almost one-third stated that incidents of WPV have made them less satisfied with their job. Suggested ideas to reduce WPV include improvement communication strategies, public outreach campaigns, and de-escalation training [13]. Hospital-based interventions include improved security and better law enforcement, including limiting the number of bystanders in the ED [12]. Recommendations include more buy-in from ED and hospital leadership and administration, as well as the police, to improve their system of reporting WPV and to provide more resources for victims of WPV from the hospital. Haddon’s matrix may be a helpful brainstorming tool for considering preventative interventions related to WPV [23]. This will provide a structure to identify factors associated with WPV and prevention interventions.

Limitations:

This study was conducted in private hospitals in India, which may differ in financial rates, patient population, and staffing resources making studies of WPV in the private system less applicable to the public sector. This study found a high incidence of ED WPV among early career staff; however, only 15.5% of respondents had more than ten years of practice experience, introducing potential selection bias.

## Conclusion

ED healthcare providers in India face higher rates of WPV, particularly verbal abuse, compared to more developed countries. There is an alarming frequency of violence from patient family members and bystanders in the ED, in contrast to studies of ED WPV in higher resource settings. Understanding this discrepancy should be a priority for healthcare institutions in India, necessitating further study. Exacerbating the issue is a lack of reporting by healthcare providers when incidents occur. Research into reporting processes and enforcement mechanisms may reveal why many incidents go unreported. Strategies to prevent WPV should be prioritized.

### Electronic supplementary material

Below is the link to the electronic supplementary material.

Supplementary Material 1

## Data Availability

The datasets used and/or analyzed during the current study are available from the corresponding author upon reasonable request.

## Abbreviations

ED: Emergency department
EM: Emergency medicine
WPV: WPV
