# Supplementary material for: Violence in the emergency department: a quantitative survey study of healthcare providers in India
Source: Int J Emerg Med. 2024 Jul 3;17:83. doi: 10.1186/s12245-024-00653-x (PMC11223359; doi:10.1186/s12245-024-00653-x)
Supplement: Supplementary file 1 — Supplementary Material 1 [file 12245_2024_653_MOESM1_ESM.pdf]

Workplace Violence Survey - 1/29/19

Survey Questions

1. What is your sex?
    a. Male
    b. Female

2. What is your age in years?
    a. < 20
    b. 20 – 30
    c. 31 – 40
    d. 41 – 50
    e. 51 – 60
    f. > 60 years

3. How many years have you been working in clinical practice (after finishing your MBBS, nursing degree, BN or other schooling)?
    a. <5
    b. 5 – 10 years
    c. 11 – 15 years
    d. 16 – 20 years
    e. More than 20 years

4. What is your job title?
    a. Nurse
    b. Resident – PGY1, PGY2, PGY3
    c. Paramedic
    d. Consultant/attending
    e. Other

5. Which of the following best describes your hospital location?
    a. Urban large city (population > 500,000)
    b. Urban small city (population < 500,000)
    c. Suburban
    d. Rural

Verbal abuse is defined as “any oral communication that is directly and specifically menacing to one’s well-being.” This does not include random swearing or other speech that has no implied intent to harm.

6. Have you ever witnessed verbal abuse in the Emergency Department?
    a. Yes
    b. No
7. IF YES - who was the abusive party? (select all the apply)
    a. Patient

b. Patient's family/Bystanders
c. Emergency Department staff member
d. Other hospital staff member
e. Specialist from another department
f. Other

8. Have you ever been verbally abused in the Emergency Department?
   a. Yes
   b. No
9. IF YES - who was the abusive party? (select all the apply)
   a. Patient
   b. Patient's family/Bystanders
   c. Emergency Department staff member
   d. Other hospital staff member
   e. Specialist from another department
   f. Other
10. How often does this kind of abuse occur in the Emergency Department?
    a. Daily
    b. Weekly
    c. Monthly
    d. Every 6 months
    e. Yearly
    f. Never
11. Have you ever reported an incident of verbal abuse?
    a. Yes
    b. No
12. If YES - To whom did you report the incident? (select all that apply)
    a. Local police
    b. Hospital police
    c. Emergency Department administrator
    d. Hospital administrator
    e. Other
13. If NO – Why didn't you report the incident?
    a. I have never experienced an incident that was serious enough to report
    b. Belief that nothing would be done even if reported
    c. Fear of retribution
    d. I have never witnessed or experienced verbal abuse in the emergency department
    e. Other

Physical abuse is defined as "any incident where there was physical contact of an unwanted nature that resulted in harm or was perceived as threatening."

14. Have you ever witnessed physical abuse in the Emergency Department?
    a. Yes
    b. No
15. IF YES – who was the abusive party (select all that apply)

a. Patient
b. Patient’s family/Bystanders
c. Emergency Department staff member
d. Other hospital staff member
e. Specialist from another department
f. Other

16. Have you ever been physically abused in the Emergency Department?
    a. Yes
    b. No
17. IF YES – who was the abusive party (select all that apply)
    a. Patient
    b. Patient’s family/Bystanders
    c. Emergency Department staff member
    d. Other hospital staff member
    e. Specialist from another department
    f. Other
18. How often does this kind of abuse occur in the Emergency Department?
    a. Daily
    b. Weekly
    c. Monthly
    d. Yearly
    e. Almost never
    f. Never
19. Have you ever reported an incident of physical abuse?
    a. Yes
    b. No
20. IF YES - To whom did you report the incident? (select all that apply)
    a. Local police
    b. Hospital police
    c. Emergency Department administrator
    d. Hospital administrator
    e. Other
21. IF NO – Why didn’t you report the incident?
    a. I have never experienced an incident that was serious enough to report
    b. Belief that nothing would be done even if reported
    c. Fear of retribution
    d. I have never witnessed or experienced physical abuse in the emergency department
    e. Other

An outside confrontation is defined as, “any unpleasant or threatening interaction with a patient or their family or bystanders that occurs after the time of providing medical care.”

22. Have you ever had an outside confrontation after a patient encounter?
    a. Yes
    b. No

23. IF YES – with whom did you have the confrontation? (select all that apply)
    a. Patient
    b. Patient's family/Bystanders
    c. Emergency Department staff member
    d. Other hospital staff member
    e. Specialist from another department
    f. Other
24. Have you ever witnessed a colleague have an outside confrontation after a patient encounter?
    a. Yes
    b. No
25. IF YES – with whom did the confrontation occur? (select all that apply)
    a. Patient
    b. Patient's family/Bystanders
    c. Emergency Department staff member
    d. Other hospital staff member
    e. Specialist from another department
    f. Other
26. How often does this kind of abuse occur as a result of encounters in the Emergency Department?
    a. Daily
    b. Weekly
    c. Monthly
    d. Yearly
    e. Almost never
    f. Never
27. Have you ever reported an incident from an outside confrontation?
    a. Yes
    b. No
28. If YES - To whom did you report the incident? (select all that apply)
    a. Local police
    b. Hospital police
    c. Emergency Department administrator
    d. Hospital administrator
    e. Other
29. IF NO – Why didn't you report the incident?
    a. I have never experienced an incident that was serious enough to report
    b. Belief that nothing would be done even if reported
    c. Fear of retribution
    d. I have never witnessed or experienced an outside confrontation
    e. Other

Stalking behavior is defined as, "unwanted or threatening behavior by the patient or someone representing the patient in a persistent manner over time."

30. Have you ever been stalked as a result of a patient encounter?

a. Yes
b. No

31. If YES – with who stalked you? (select all that apply)
    a. Patient
    b. Patient's family/Bystanders
    c. Emergency Department staff member
    d. Other hospital staff member
    e. Specialist from another department
    f. Other
32. How often does this kind of abuse occur as a result of encounters in the Emergency Department?
    a. Daily
    b. Weekly
    c. Monthly
    d. Yearly
    e. Almost never
    f. Never
33. Have you ever reported an incident of being stalked?
    a. Yes
    b. No
34. If YES - To whom did you report the incident? (select all that apply)
    a. Local police
    b. Hospital police
    c. Emergency Department administrator
    d. Hospital administrator
    e. Other
35. IF NO – Why didn't you report the incident?
    a. I have never experienced an incident that was serious enough to report
    b. Belief that nothing would be done even if reported
    c. Fear of retribution
    d. I have never experienced stalking
    e. Other

36. If you did report an incident of workplace violence (either verbal, physical, outside confrontation, or stalking), were you supplied with resources (select all that apply)? If you have never reported an incident of workplace violence, skip this question.
    a. Yes – the hospital supplied resources
    b. Yes – police supplied resources
    c. Yes – my family/friends helped me get resources
    d. No resources were made available

37. Have you ever experienced an incident where a weapon was brandished against you?
    a. Yes
    b. No

38. Have incidents of workplace violence made you less satisfied with your job?
    a. Yes
    b. No
39. Have incidents of workplace violence ever made you afraid to go to work?
    a. Yes
    b. No
40. Have you ever missed work due to a violent incident?
    a. Yes
    b. No
41. Have you ever lost sleep at night as a result of a violent incident at work?
    a. Yes
    b. No
42. As a result of workplace violence have you have ever done any of the following? (select all that apply)
    a. Considered leaving your current hospital position
    b. Left a previous hospital position
    c. Considered leaving the practice of emergency medicine
    d. Sought legal counseling/support?
    e. Sought psychological counseling/support
    f. Sought to obtain personal protection (weapons, pepper spray, etc.)
    g. Sought help from the police
43. Which of the following security measures are available within your emergency department? (select all that apply)
    a. Hospital security assigned permanently to the Emergency Department
    b. Hospital security that can be called to the Emergency Department, but are not stationed there permanently
    c. Armed security officers
    d. Unarmed security officers
    e. Police
    f. Screening visitors for weapons
    g. Walk through metal detectors
    h. Hand held metal detectors
    i. No security staff

44. Has the COVID-19 pandemic affected the incidence of workplace violence in your Emergency Department?
    a. Yes
    b. No

45. If YES – how?
    a. There is more workplace violence
    b. There is less workplace violence

46. If there is MORE workplace violence – which kinds of violence have increased? (select all that apply)
    a. There is more verbal abuse
    b. There is more physical abuse
    c. There are more outside confrontations
    d. There is more stalking behavior

47. If there is LESS workplace violence – which kinds of violence of decreased? (select all that apply)
    a. There is less verbal abuse
    b. There is less physical abuse
    c. There are fewer outside confrontations
    d. There is less stalking behavior
